# Semaglutide and Tirzepatide in a Remote Weight Management Program: 12-Month Retrospective Observational Study

**DOI:** 10.2196/81912

**Published:** 2025-09-12

**Authors:** Rebecca Richards, William Lunt, Michael Whitman, Giulia Spaltro, Rachel Hall

**Affiliations:** 1 Second Nature London United Kingdom

**Keywords:** obesity, weight management, glucagonlike peptide-1 receptor agonist, GLP-1RA, semaglutide, digital health, remote health care delivery, telemedicine, behavioral intervention, weight loss

## Abstract

**Background:**

Obesity affects >890 million adults worldwide, and traditional lifestyle interventions often lack long-term success. While glucagonlike peptide-1 receptor agonists (GLP-1RAs) have shown strong weight loss outcomes, access to specialist care is limited by cost and capacity.

**Objective:**

This study evaluated the effectiveness, feasibility, acceptability, and potential cost-effectiveness of a 12-month remote GLP-1RA–supported weight management program, comparing outcomes between tirzepatide and semaglutide.

**Methods:**

This retrospective analysis included 339 participants (n=278, 82% women) who completed a 12-month remote weight management program using either tirzepatide (n=209, 61.7%) or semaglutide (n=130, 38.3%) between February and June 2024. The program combined medication, app-based behavioral support, coaching from registered dietitians and nutritionists, and clinical oversight. It featured 5 phases with evidence-based behavior change techniques, monthly monitoring, and safety protocols. Primary outcomes were mean weight change and proportions achieving ≥10% and ≥15% weight loss. Secondary outcomes included behavior changes, side effects, acceptability, feasibility, and estimated cost-effectiveness compared to National Health Service care.

**Results:**

Mean weight change at 12 months was −22.9 kg (–22.1% of baseline weight, SD 8%; *P*<.001) in the tirzepatide cohort and −18.1 kg (–17.1% of baseline weight, SD 8.1%; *P*<.001) in the semaglutide cohort. Achievement of ≥10% weight loss occurred in 95.2% (199/209) of participants using tirzepatide and 83.1% (108/130) of participants using semaglutide, whereas ≥15% weight loss was achieved by 83.7% (175/209) and 56.2% (73/130) of the participants, respectively. The proportion of inactive participants (no weekly exercise) decreased substantially in both cohorts (tirzepatide: 31/209, 14.8% to 14/209, 6.7%; semaglutide: 29/130, 22.3% to 7/130, 5.4%; *P*<.001). Side effects decreased significantly over the 12-month period, with participants who reported no side effects increasing from 41.6% (87/209) to 60.3% (126/209; *P*<.001) in the tirzepatide cohort and from 53.8% (70/130) to 67.7% (88/130) in the semaglutide cohort (*P*=.02), whereas common initial side effects, including constipation, nausea, and fatigue, showed significant reductions (*P*<.001). Economic modeling suggested a 60% to 70% cost saving compared to specialist weight management services and a 10% to 60% cost saving compared to primary care in the National Health Service.

**Conclusions:**

This real-world evaluation demonstrates that remotely delivered, GLP-1RA–supported weight management programs can achieve weight loss outcomes that align closely with clinical trial results while potentially reducing health care costs by 10% to 70% compared to traditional UK services. Both the tirzepatide and semaglutide cohorts exceeded clinically significant weight loss thresholds with acceptable safety profiles and positive behavior changes. These findings support the feasibility and effectiveness of digital delivery models for expanding access to specialist obesity treatment within resource-constrained health care systems, with outcomes that compare favorably to pharmacological intervention alone.

## Introduction

### Background

Worldwide, >2.5 billion adults are overweight. Of this number, >890 million, or approximately 1 in 10 adults, are living with obesity [1]. Obesity is defined as a chronic, systemic illness that is underpinned by excess adiposity that can lead to severe organ dysfunction and life-threatening complications (eg, stroke, cardiovascular disease, type 2 diabetes, depression, and certain types of cancer) [1,2]. In the United Kingdom, obesity is expected to cost the National Health Service (NHS) nearly £12 billion (US $16.1 billion) in 2025, with total costs (NHS, individual, and wider societal) projected to reach >£73 billion (US $98.1 billion) [3,4].

While lifestyle interventions have historically formed the foundation of obesity treatment, their long-term effectiveness varies considerably, with more than half of lost weight typically regained within 2 years [5]. Following the success of glucagonlike peptide-1 receptor agonists (GLP-1RAs) in the treatment of type 2 diabetes mellitus, the Semaglutide Treatment Effect in People With Obesity (STEP) trials have demonstrated significant potential for this medication in weight management [6,7]. Subsequent trials of semaglutide, tirzepatide, and retratutide have demonstrated weight reductions of 14.9%, 20.9%, and 24.2% over 68 to 88 weeks, respectively. However, significant weight regain occurs following treatment discontinuation [8-11]. In both the STEP-1 and STEP-4 (semaglutide) trials and the SURMOUNT-4 (tirzepatide) trial, participants regained approximately two-thirds of the lost weight within 1 year of discontinuing treatment [9,12,13]. To improve efficacy in obesity care and reduce the risk of weight regain, the National Institute for Health and Care Excellence (NICE) recommended tirzepatide for indefinite use in obesity management, in conjunction with lifestyle interventions (NICE Technology Appraisal TA1026) [14].

In line with the NICE Technology Appraisal TA1026, the NHS Long Term Plan, and NHS England’s interim commissioning guidance for tirzepatide implementation [14-16], there is growing pressure on primary care, as well as in specialist weight management services (SWMSs), to prescribe GLP-1RAs. Combined with existing waiting lists, limited budgets, and reduced staffing, both primary and secondary care have struggled to meet the mandated 90- and 180-day NICE implementation timelines [16]. NHS England is commissioning a national pilot Behavioural Support for Obesity Prescribing service to support tirzepatide prescribing—as mandated by the NICE guidelines—but the impact and utility of this service vary significantly among integrated care boards (ICBs) [16].

Evidence demonstrates that behavior change, alongside GLP-1RA medication, delivers greater weight loss over a 12-month period than medication alone [17]. Combined behavioral and pharmacological approaches have achieved a 19.1% reduction in body weight as compared to the 14.9% observed in pharmacological clinical trials [8,17]. Furthermore, initial UK-based cost modeling suggests that remotely delivered services are more scalable and significantly less expensive when delivered in primary care [14]. Following recent commissioning guidance changes, the evaluation of how remote services integrate with existing weight management services to deliver tirzepatide is essential.

### Objectives

This 12-month retrospective observational analysis examined the effectiveness, feasibility, acceptability, and potential cost-effectiveness of Second Nature’s remotely delivered weight management program supported by GLP-1RAs. This study also aimed to compare the outcomes of semaglutide and tirzepatide within a remote care setting.

## Methods

For the purpose of reporting this evaluation, we have followed and used NHS England’s evaluation toolkit checklist [18].

### Participants

This service evaluation was conducted within Second Nature’s commercial digital health platform, which operates as a private weight management service (independent of NHS infrastructure) in the United Kingdom. Participants were recruited via various digital marketing channels (eg, Google, Facebook, and Instagram) or through word-of-mouth referrals. No specific demographic or geographic parameter was targeted. All participants were enrolled via self-referral completing online eligibility questionnaires via the Second Nature website and app.

In July 2025, retrospective data were obtained for all participants who (1) began either the semaglutide- or tirzepatide-supported intervention during the period from February 2024 to June 2024; (2) completed the intervention; and (3) provided responses to the 1-, 3-, 6-, and 12-month surveys. All participants paid between £229 (US $310) and £299 (US $400) per month depending on the dose of tirzepatide or semaglutide prescribed.

Adults aged 18 to 75 years with weight-related comorbidities were eligible, with BMI thresholds of ≥35 kg/m^2^ for tirzepatide and ≥30 kg/m^2^ for semaglutide (tier 3 referral criteria for BMI: 30.0-34.9 kg/m^2^). BMI thresholds were reduced by 2.5 kg/m^2^ for participants from South Asian, Chinese, other Asian, Middle Eastern, Black African, or African-Caribbean backgrounds [14,19].

Users had to possess access and demonstrate capability to use smartphone or tablet devices to be considered eligible for participation. Several exclusion criteria for both medications were established: individuals with current or previous eating disorder diagnoses; those who were pregnant, nursing, or actively seeking to become pregnant; participants with known allergies to any medication components or excipients; and those currently using specific medications (including diabetes treatments or drugs associated with the aforementioned exclusion conditions). Further exclusions applied to individuals presenting with any of the following medical conditions: thyroid cancer (current or historical), active malignancy, inflammatory bowel conditions (including Crohn’s disease and ulcerative colitis), celiac disease, chronic malabsorption syndrome, pancreatitis, hepatic impairment, renal disease, cardiac failure, multiple endocrine neoplasia type 2, gallbladder disorders, or diabetic retinopathy.

As we have previously reported attrition—which, for a private intervention, is usually heavily weighted toward costs [17]—we wanted to focus on showing real-world effectiveness and medication tolerability and attaining the largest sample size for the most accurate effect size analysis. To do this, we self-selected participants who both completed the program and provided survey responses at months 1, 3, 6, and 12.

### Intervention

This intervention builds on the previously described Second Nature digital program, which was developed by an in-house multidisciplinary team following NICE guidance for obesity management and behavior change principles [14,17,20-23]. The program consists of 5 phases (prepare, adapt, grow, progress, and momentum) spanning ≥68 weeks, delivering evidence-based behavior change techniques through daily educational content covering nutrition, medication management, sleep, mental well-being, and physical activity [24]. The web and mobile platform integrates self-monitoring tools for weight, sleep, and activity tracking via connected devices, whereas health coaches provide personalized support through one-to-one messaging, establish individualized weight and activity goals, and proactively engage participants who have not accessed the app for ≥4 days. Participants receive automated feedback through weekly progress graphs and health change summaries, with mandatory monthly surveys and weight measurements prompted by the app’s notification system.

### Medication

Participants chose between semaglutide or tirzepatide at enrollment, with medication-specific protocols and educational resources provided through the Second Nature app. Tirzepatide users received more intensive monitoring (weekly surveys and 3-5–day pharmacist intervention) compared to semaglutide users (monthly monitoring and 7-day intervention) due to increased side effect profiles [25]. Safety monitoring included monthly pharmacist-reviewed digital surveys, Bluetooth-connected weight tracking, and a tiered adverse event protocol with health coach response within 24 hours and pharmacist escalation within 4 hours for urgent concerns.

### Effectiveness Measures

The primary outcome was the mean weight change, recorded between days 330 and 420 (approximately months 11 to 13) after program initiation, assessed in both kilograms and as a percentage of baseline weight. Secondary measures included the proportion of participants achieving ≥10% and ≥15% weight loss, along with monthly weight change data corresponding to each survey point for both pharmacological interventions.

Participants were provided with wireless weighing scales to share weight data with Second Nature. To ensure accuracy, they were instructed to weigh themselves on a firm, flat surface in the morning after using the restroom and at consistent weekly intervals. A validation algorithm accepted only readings within defined thresholds based on previous measurements and the time elapsed since the last entry, helping filter out anomalies and monitor for sudden changes in weight. If irregular readings were detected—such as when someone else in the household used the scale—participants received email notifications, and the data were not stored.

### Cost Measures

We have previously reported the cost-effectiveness of a semaglutide-supported intervention compared to NHS SWMSs [17]. Given that tirzepatide is now recommended for use in primary care and specialist service settings, we conducted a cost comparison of the tirzepatide-supported intervention against both NHS SWMSs and nonspecialist primary care weight management services. Cost data for both services were obtained from NICE Technology Appraisal TA1026 resource impact templates [14].

A range of confidential costs for Second Nature’s program were used, including the lower-intensity, lower-cost private service (evaluated in this study) and a higher-intensity model assuming that NHS delivery would require delivery to more complex patients based on the NHS interim commissioning guidance (BMI of >40 kg/m^2^ and 4 comorbidities for the phase 1 cohort) [14]. To maintain commercial confidentiality, costs were presented as percentage reductions rounded to 10% intervals using relative cost methodology. We computed percentage variations between the intervention and primary and secondary care standard approaches outlined in the NICE health technology evaluation 14 and tirzepatide Technology Appraisal documents [14,26]. All costs were standardized to per-participant amounts over a 12-month treatment period. The analysis focused exclusively on direct intervention costs excluding potential long-term health benefit savings.

Cost comparisons were conducted using simple cost analysis between the tirzepatide-supported intervention and both NHS standard care models (SWMSs and nonspecialist primary care). Key parameters included 6.2% total eligibility (NICE default), 30% digital service uptake, and 75% medication initiation rate based on internal data from Second Nature’s NHS services [17,26]. On the basis of these parameters, 1395 participants per 100,000 population were expected to enroll. Percentage cost differences were calculated as ([comparator cost − intervention cost]/comparator cost) × 100 to determine potential savings. Real-world GBP costs were calculated as number of participants (1395) × lower-range cost and number of participants (1395) × higher-range cost to provide an expected cost of delivery. This expected cost of delivery was then multiplied by the expected cost saving percentage (highest range number × lowest cost saving and lowest range number × highest cost saving) to provide an expected saving in GBP.

### Behavior Changes

Web-based surveys administered at months 1, 3, 6, and 12 of the program assessed lifestyle-related behavioral modifications, encompassing cooking frequency, fruit and vegetable consumption, and physical activity levels.

Meal preparation behaviors were examined using the following question**―**“How many times do you cook each week on average?”―with responses documented as weekly cooking session numbers.

Daily vegetable and fruit consumption was evaluated using the following question**―**“How many different vegetables or fruits do you eat a day on average?”―with numerical responses indicating the average number of distinct items consumed daily.

Physical activity assessment used the following question―“How many times do you exercise each week on average?”―requiring participants to report their average weekly exercise session frequency.

### Acceptability: Participant Experience

Free-text responses to the monthly survey question―“How have you found the last few weeks of the program?”―underwent content analysis. MW and WL coded and organized responses into higher-level categories for each question at every time point. These analytical frameworks underwent refinement following protocols established in previous research, resulting in 9 higher-level categories that encompassed key themes within participants’ experiences [21]. To capture the complexity of participant experiences, individual responses could be classified across multiple themes. For instance, a participant’s free-text response of *Ok* to “How have you found the last few weeks on the program?” would be categorized as a *neutral experience*.

### Feasibility: Side Effects

Mandatory web-based check-in surveys were emailed to participants at 1, 3, 6, and 12 months to gather medication side effect information. A drop-down menu allowed for the selection from 15 possible side effects, *None*, or *Other*. Selecting *Other* required a mandatory free-text description.

### Statistical Analysis

Analyses were conducted using the R statistical software (R Foundation for Statistical Computing). For effectiveness, we calculated mean weight change and SD and evaluated statistical significance using paired 2-tailed *t* tests (*P*<.05) plus proportions achieving ≥10% and ≥15% weight loss thresholds. Statistical analysis included descriptive statistics for baseline characteristics and primary weight change outcomes.

Monthly behavior changes, side effects, and participant experiences were analyzed as proportions of total survey respondents. For each behavioral subcategory, we conducted 2-proportion *z* tests comparing the proportion of participants in that specific category at each follow-up time point (months 3, 6, and 12) against the baseline proportion (month 1). Separate analyses were conducted for each medication group (tirzepatide and semaglutide). Given the multiple subcategory comparisons within each behavioral domain, we applied the Bonferroni correction to control the familywise error rate.

### Ethical Considerations

This evaluation met Health Research Authority and Medical Research Council service evaluation criteria [26]**―**it assessed current care using existing, anonymized data from an established intervention; did not involve any change to standard practice; and did not involve randomization or a control group. The Health Research Authority explicitly states that service evaluation is considered part of usual professional practice and is exempt from research ethics committee oversight [27]. This study analyzed anonymized, non-NHS data that could not identify individuals, thus not requiring ethics approval (Multimedia Appendix 1); however, the data were not initially collected with a view to use them in this research. Participants were not compensated for taking part and consented to research use of anonymized data through acceptance of the privacy policy at registration. This evaluation adhered to the General Data Protection Regulation, maintaining participants’ right to request data deletion.

## Results

### Baseline Characteristics

A total of 339 participants were included in the analysis. Of these, 82% (278/339) were women, and the average age was 48.4 (SD 10.7) years. The mean baseline BMI was 36.7 (SD 7.3) kg/m^2^ (Table 1).

In the tirzepatide cohort (209/339, 61.7%), 82.8% (173/209) of the participants were women, with an average age of 48.2 (SD 10.6) years. The mean baseline BMI was 36.4 (SD 6.8) kg/m^2^ (Table 1).

In the semaglutide cohort (130/339, 38.3%), 80.8% (105/130) of the participants were women. The average age was 48.8 (SD 10.9) years. The mean baseline BMI was 37.0 (SD 7.9) kg/m^2^ (Table 1).

**Table 1:** Baseline characteristics for all participants, participants in the tirzepatide cohort, and participants in the semaglutide cohort (N=339).

Characteristic	All participants	Tirzepatide cohort participants (n=209)	Semaglutide cohort participants (n=130)
Age (y), mean (SD)	48.4 (10.7)	48.2 (10.6)	48.8 (10.9)
Female sex, n (%)	278 (82)	173 (82.8)	105 (80.8)
Baseline weight (kg), mean (SD)	103.8 (23.6)	103.5 (22.6)	104.1 (25.3)
Baseline BMI (kg/m^2^), mean (SD)	36.7 (7.3)	36.4 (6.8)	37.0 (7.9)

### Effectiveness

#### Weight Change

**Figure 1:**
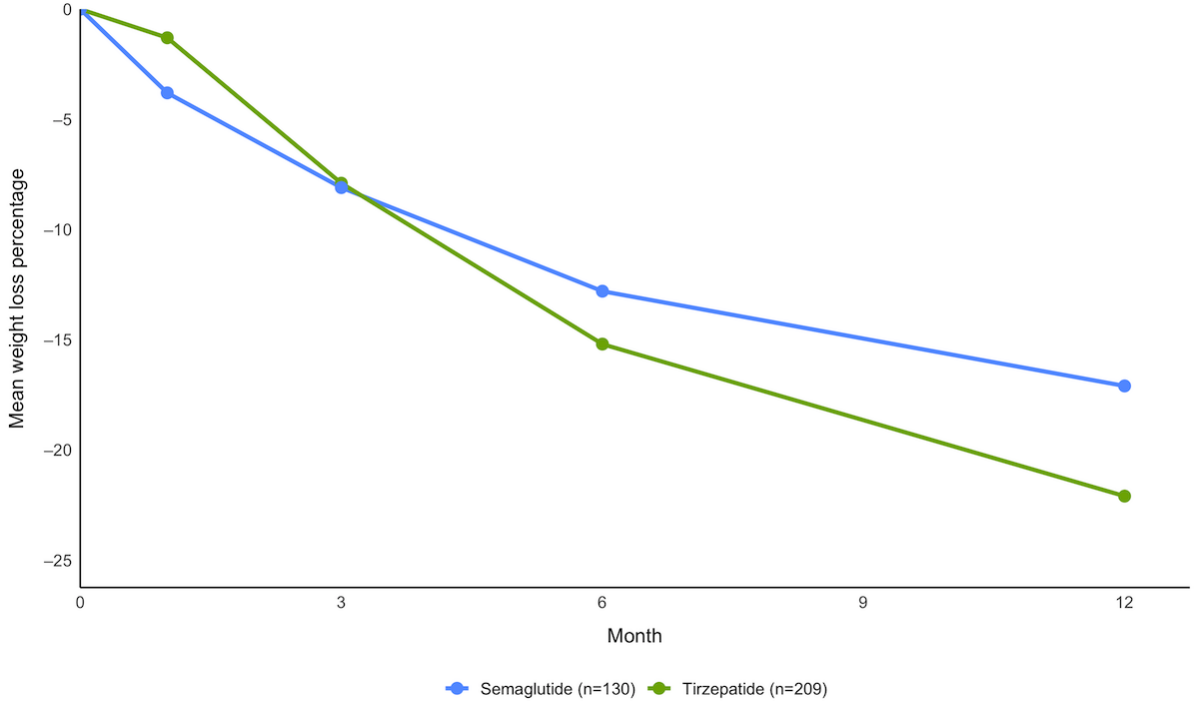
Weight change percentage over 12 months (semaglutide vs tirzepatide).

The mean weight change observed in the tirzepatide cohort (209/339, 61.7%) over the 12 months was −22.9 (SD 9.9; *P*<.001) kg, or −22.1% (SD 8%) of participants’ starting weight. A total of 95.2% (199/209) of the participants achieved ≥10% weight loss, and 83.7% (175/209) achieved ≥15% weight loss (Figure 1 and Table 2).

In the semaglutide cohort (130/339, 38.3%), the mean weight change observed over the 12-month period was −18.1 (SD 12.9; *P*<.001) kg, or −17.1% (SD 8.1%) of participants’ starting weight. A total of 83.1% (108/130) of the participants achieved ≥10% weight loss, and 56.2% (73/130) achieved ≥15% weight loss (Figure 1, Table 2).

**Table 2:** Mean weight change percentage achieved in months 1, 3, 6, and 12 by molecule.

Month	Tirzepatide cohort (n=209; %), mean (SD)	Semaglutide cohort (n=130; %), mean (SD)
1	−1.3 (4.1)	−3.8 (14.1)
3	−7.9 (4.8)	−8.1 (14.2)
6	−15.2 (5.8)	−12.8 (14.6)
12	−22.1 (9.8)	−17.1 (16.7)

#### Cost Compared to Usual Care

The cost of delivering behavior change support in a secondary care SWMS (ie, delivered by a multidisciplinary team) was estimated by the external assessment group for the NICE health technology evaluation 14 guidance to be £1796 (US $2413.81) per patient per year [20]. We have previously evaluated that the Second Nature service could provide 60% to 70% cost savings when compared to SWMSs [17].

The cost of delivering behavior change support in a primary care or community-based, nonspecialist service has been estimated by the NICE to be between £868.21 (US $1166.87) and £1239.21 (US $1665.49) per patient per year [14,28]. Second Nature’s model could deliver 10% to 60% cost savings compared to the NICE’s primary care modeling.

For an expected 1395 enrollments per 100,000 persons, the total cost for delivering a service would be £1.2 to 1.7 million (US $1.6-$2.3 million), meaning that Second Nature’s model could save between £120,000 (US $161,279; 10%) and £1,020,000 (US $1,370,870; 60%) per 100,000 individuals.

### Behavior Changes

When asked about fruit and vegetable consumption, over half (114/209, 54.5%) of the participants in the tirzepatide cohort consistently reported consuming 3 to 4 different items per day over the 12-month period except for month 3 (Table 3). This trend was also observed in the semaglutide cohort; however, this group consistently had a higher proportion of respondents (112/209, 53.6% of the tirzepatide cohort vs 73/130, 56.2% of the semaglutide cohort in month 1) and had no reduction in month 3.

Regarding exercise frequency, the proportion of participants in the tirzepatide cohort who were already exercising 3 to 4 times per week generally increased over the course of the study (from 73/209, 34.9% in month 1 to 90/209, 43.1% in month 12); however, this trend stopped in month 6. This trend was also observed in the semaglutide cohort (from 43/130, 33.1% in month 1 to 57/130, 43.8% in month 12). Across both cohorts, the percentage of respondents who never exercised each week also decreased, with the largest, statistically significant change observed in the semaglutide cohort (from 29/130, 22.3% in month 1 to 7/130, 5.4% in month 12; *P*<.001).

Across both cohorts, a decrease in cooking frequency was observed over the course of 12 months. In the tirzepatide cohort, the percentage of respondents who cooked every day decreased from 30.1% (63/209) in month 1 to 19.6% (41/209) in month 12, whereas in the semaglutide cohort, it decreased significantly from 41.5% (54/130) to 23.1% (30/130; *P*=.03). There was minimal change in those who never cooked across the 12 months; however, a substantial increase in the percentage of respondents who cooked *a few times a week* was observed in the tirzepatide cohort, changing from 24.4% (51/209) to 32.5% (68/209). In the semaglutide cohort, a similar trend was also observed, changing from 23.1% (30/130) in month 1 to 30% (39/130) in month 12.

**Table 3:** Responses to the lifestyle-related behavioral survey by month.

Category and subcategory		Month 1, n (%)		Month 3, n (%)		Month 6, n (%)		Month 12, n (%)	
		Tirzepatide cohort (n=209)	Semaglutide cohort (n=130)	Tirzepatide cohort (n=209)	Semaglutide cohort (n=130)	Tirzepatide cohort (n=209)	Semaglutide cohort (n=130)	Tirzepatide cohort (n=209)	Semaglutide cohort (n=130)
**Fruit and vegetable consumption (items per day)**									
	0	0 (0)	3 (2.3)	0 (0)	1 (0.8)	2 (1)	1 (0.8)	0 (0)	1 (0.8)
	1-2	36 (17.2)	17 (13.1)	50 (23.9)	23 (17.7)	44 (21.1)	26 (20)	40 (19.1)	29 (22.3)
	3-4	112 (53.6)	73 (56.2)	97 (46.4)	69 (53.1)	114 (54.5)	74 (56.9)	114 (54.5)	72 (55.4)
	≥5	61 (29.2)	37 (28.5)	62 (29.7)	37 (28.5)	49 (23.4)	29 (22.3)	55 (26.3)	28 (21.5)
**Exercise (times per week)**									
	0	31 (14.8)	29 (22.3)	25 (12)	13 (10)	17 (8.1)	9 (6.9)	14 (6.7)^a^	7 (5.4)^b^
	1-2	85 (40.7)	47 (36.2)	87 (41.6)	51 (39.2)	105 (50.2)	54 (41.5)	83 (39.7)	52 (40)
	3-4	73 (34.9)	43 (33.1)	75 (35.9)	52 (40)	67 (32.1)	54 (41.5)	90 (43.1)	57 (43.8)
	≥5	20 (9.6)	11 (8.5)	22 (10.5)	14 (10.8)	20 (9.6)	13 (10)	22 (10.5)	14 (10.8)
**Cooking frequency**									
	Never	4 (1.9)	0 (0)	3 (1.4)	1 (0.8)	1 (0.5)	0 (0)	2 (1)	1 (0.8)
	Less than once a week	2 (1)	2 (1.5)	2 (1)	5 (3.8)	7 (3.3)	6 (4.6)	5 (2.4)	1 (0.8)
	Once a week	4 (1.9)	3 (2.3)	8 (3.8)	4 (3.1)	13 (6.2)	4 (3.1)	11 (5.3)	8 (6.2)
	Few times a week	51 (24.4)	30 (23.1)	73 (34.9)	28 (21.5)	67 (32.1)^c^	41 (31.5)	68 (32.5)^d^	39 (30)
	Nearly every day	85 (40.7)	41 (31.5)	77 (36.8)	58 (44.6)	81 (38.8)	55 (42.3)	82 (39.2)	51 (39.2)
	Every day	63 (30.1)	54 (41.5)	46 (22)	34 (26.2)	40 (19.1)	24 (18.5)^e^	41 (19.6)^d^	30 (23.1)^d^

^a^*P*=.04.

^b^*P*<.001.

^c^*P*<.001.

^d^*P*=.03.

^e^*P*=.02.

### Acceptability: Participant Experience

The participant responses were organized into 9 thematic categories: change in appetite, illness, intervention benefits, negative experience, neutral experience, other, other priorities, positive experience, and slow progress.

At month 1, across both cohorts, most participants reported positive experiences (130/209, 62.2% and 90/130, 69.2% for the tirzepatide and semaglutide cohorts, respectively). This declined significantly to an approximate third by month 12 (66/209, 31.6% and 51/130, 39.2% for the tirzepatide and semaglutide cohorts, respectively; *P*<.001). Negative experiences did not noticeably increase; however, neutral experiences significantly increased from 12.9% (27/209) in the tirzepatide cohort and 15.4% (20/130) in the semaglutide cohort at month 1 to 52.2% (109/209) and 50% (65/130) by month 12, respectively (Table 4; *P*<.001).

There was a trend in both change in appetite and intervention benefits in that, across both the semaglutide and tirzepatide cohorts, a reduction in reporting was observed. A total of 12.3% (16/130) of semaglutide users reported a change in appetite in month 1, but only 0.8% (1/130) did so in month 12. In total, 21.1% (44/209) of tirzepatide users reported this effect in month 1, but only 1.4% (3/209) did so in month 12. Both cohorts showed a significant reduction (*P*<.001). Intervention benefits decreased in reporting levels from 20% (26/130) to 3.1% (17/130) from month 1 to 12 (*P*<.001) in the semaglutide cohort, whereas in the tirzepatide cohort, it dropped from 12.4% (26/209) to 6.7% (14/209) over the same time frame.

**Table 4:** Percentage of cohort participants reporting different experiences throughout by program month.

Experience	Month 1, n (%)		Month 3, n (%)		Month 6, n (%)		Month 12, n (%)	
	Tirzepatide cohort (n=209)	Semaglutide cohort (n=130)	Tirzepatide cohort (n=209)	Semaglutide cohort (n=130)	Tirzepatide cohort (n=209)	Semaglutide cohort (n=130)	Tirzepatide cohort (n=209)	Semaglutide cohort (n=130)
Change in appetite	44 (21.1)	16 (12.3)	12 (5.7)^a^	10 (7.7)	7 (3.3)^a^	4 (3.1)	3 (1.4)^a^	1 (0.8)^a^
Illness	1 (0.5)	1 (0.8)	3 (1.4)	3 (2.3)	2 (1)	3 (2.3)	0 (0)	0 (0)
Intervention benefits	26 (12.4)	26 (20)	29 (13.9)	17 (13.1)	10 (4.8)	14 (10.8)	14 (6.7)	4 (3.1)
Negative experience	0 (0)	1 (0.8)	0 (0)	0 (0)	0 (0)	1 (0.8)	0 (0)	0 (0)
Neutral experience	27 (12.9)	20 (15.4)	41 (19.6)	24 (18.5)	68 (32.5)^a^	42 (32.3)^a^	109 (52.2)^a^	65 (50)^a^
Other	21 (10)	9 (6.9)	20 (9.6)	10 (7.7)	35 (16.7)	2 (1.5)	13 (6.2)	6 (4.6)
Other priorities	6 (2.9)	4 (3.1)	10 (4.8)	2 (1.5)	6 (2.9)	1 (0.8)	4 (1.9)	3 (2.3)
Positive experience	130 (62.2)	90 (69.2)	98 (46.9)^a^	70 (53.8)	83 (39.7)^a^	65 (50)^a^	66 (31.6)^a^	51 (39.2)^a^
Reduced effectiveness or slow progress	0 (0)	1 (0.8)	0 (0)	0 (0)	1 (0.5)	0 (0)	0 (0)	0 (0)

^a^*P*<.001.

### Feasibility: Side Effects

In the first month for the tirzepatide cohort, the most commonly reported side effects were constipation (61/209, 29.2%), feeling sick (58/209, 27.8%), and feeling more tired than usual (45/209, 21.5%), whereas 41.6% (87/209) reported no side effects at this stage. After 12 months, 60.3% (126/209; *P*<.05) of participants reported no side effects, and there was a significant decrease in the prevalence of the most common side effects (constipation: 27/209, 12.9% and *P*<.001; feeling sick: 22/209, 10.5% and *P*<.001; feeling more tired than usual: 17/209, 8.1% and *P*<.001) but an increase in hair loss (20/209, 9.6%; *P*<.001; Table 5).

These effects were similarly observed in the semaglutide cohort, with constipation (41/130, 31.5%), feeling sick (41/130, 31.5%), and feeling more tired than usual (32/130, 24.6%) decreasing in prevalence. In month 12, a total of 67.7% (88/130) of the participants reported no side effects, 15.4% (20/130) reported constipation, 3.1% (4/130) reported feeling sick (*P*<.001), 3.1% (4/130) reported feeling more tired than usual (*P*<.001), and 5.4% (7/130) reported hair loss (Table 5).

**Table 5:** Percentage of cohort participants reporting side effects by molecule by survey month.

Side effect	Month 1, n (%)		Month 3, n (%)		Month 6, n (%)		Month 12, n (%)	
	Tirzepatide cohort (n=209)	Semaglutide cohort (n=130)	Tirzepatide cohort (n=209)	Semaglutide cohort (n=130)	Tirzepatide cohort (n=209)	Semaglutide cohort (n=130)	Tirzepatide cohort (n=209)	Semaglutide cohort (n=130)
Constipation	61 (29.2)	41 (31.5)	45 (21.5)	40 (30.8)	42 (20.1)	23 (17.7)	27 (12.9)^a^	20 (15.4)
Diarrhea	18 (8.6)	13 (10)	20 (9.6)	14 (10.8)	26 (12.4)	7 (5.4)	13 (6.2)	7 (5.4)
Feeling anxious	5 (2.4)	2 (1.5)	4 (1.9)	1 (0.8)	1 (0.5)	3 (2.3)	4 (1.9)	1 (0.8)
Feeling dizzy	12 (5.7)	6 (4.6)	7 (3.3)	5 (3.8)	2 (1)	2 (1.5)	6 (2.9)	5 (3.8)
Feeling more tired than usual	45 (21.5)	32 (24.6)	42 (20.1)	26 (20)	29 (13.9)	23 (17.7)	17 (8.1)^b^	4 (3.1)^b^
Feeling sick	58 (27.8)	41 (31.5)	59 (28.2)	46 (35.4)	42 (20.1)	14 (10.8)^b^	22 (10.5)^b^	4 (3.1)^b^
Hair loss	3 (1.4)	2 (1.5)	6 (2.9)	1 (0.8)	22 (10.5)^b^	4 (3.1)	20 (9.6)^b^	7 (5.4)
Headaches	16 (7.7)	20 (15.4)	9 (4.3)	18 (13.8)	5 (2.4)	3 (2.3)^b^	5 (2.4)	3 (2.3)^b^
Heartburn or indigestion	37 (17.7)	11 (8.5)	37 (17.7)	27 (20.8)	21 (10)	11 (8.5)	10 (4.8)^b^	7 (5.4)
No side effects	87 (41.6)	70 (53.8)	82 (39.2)	41 (31.5)^b^	92 (44)	70 (53.8)	126 (60.3)^b^	88 (67.7)
None of the above	4 (1.9)	3 (2.3)	4 (1.9)	1 (0.8)	4 (1.9)	3 (2.3)	5 (2.4)	2 (1.5)
Reactions in the injection site	10 (4.8)	2 (1.5)	12 (5.7)	0 (0)	25 (12)	1 (0.8)	11 (5.3)	2 (1.5)
Severe stomach pain	0 (0)	1 (0.8)	0 (0)	1 (0.8)	1 (0.5)	1 (0.8)	0 (0)	1 (0.8)
Tender or swollen stomach	2 (1)	1 (0.8)	1 (0.5)	2 (1.5)	2 (1)	1 (0.8)	0 (0)	0 (0)
Vomiting	5 (2.4)	2 (1.5)	10 (4.8)	7 (5.4)	6 (2.9)	3 (2.3)	5 (2.4)	1 (0.8)

^a^*P*=.002.

^b^*P*<.001.

## Discussion

### Principal Findings

This 12-month retrospective observational analysis examined the effectiveness, feasibility, acceptability, and potential cost-effectiveness of Second Nature’s remotely delivered weight management program supported by GLP-1RAs. This study also aimed to compare the outcomes of semaglutide and tirzepatide within a remote care setting.

Users achieved substantial and clinically meaningful weight loss over 12 months, with participants in both the tirzepatide and semaglutide cohorts losing more weight over comparable periods than via pharmacological intervention alone [8-11]. In both cohorts, there was a significant decrease in the prevalence of the most common side effects—constipation, feeling more tired than usual, and feeling sick. However, there was an increase in hair loss. Accompanying this was a significant decrease in positive experiences and a significant increase in neutral experiences in both cohorts. In addition, reports of appetite changes and intervention benefits decreased markedly over the 12-month period.

Given the developing commissioning landscape of weight management within the NHS, our findings suggest that Second Nature’s intervention could result in a 60% to 70% reduction in costs compared to secondary care SWMSs and up to a 60% reduction compared to primary care and community-based services. At a population level, this translates to potential savings of £120,000 to £1,020,000 (US $161,279-$1,370,870) per 100,000 individuals. These findings indicate that remotely delivered interventions could provide substantial cost savings for ICBs during commissioning.

Comparing between GLP-1RAs, the tirzepatide cohort achieved a mean weight change of −22.9 kg (−22.1% of starting weight, SD 8%), whereas those in the semaglutide cohort lost −18.1 kg (−17.1% of starting weight, SD 8.1%) over a 12-month period (Figure 1). These outcomes substantially exceed the clinically significant threshold of 10% weight loss, with 95.2% (199/209) of tirzepatide participants and 83.1% (108/130) of semaglutide participants achieving this milestone.

### Comparison With Previous Work

#### Weight Change Outcomes

Significant differences in study design limit direct comparisons with randomized controlled trials; however, the results remain consistent with both results of clinical trials and real-world data on GLP-1RA therapy. The tirzepatide group saw a −22.1% mean weight change, closely matching results from the SURMOUNT-1 and SURMOUNT-5 trials (−20.9% and −20.2%, respectively) [10,28]. The semaglutide group achieved −17.1% mean weight loss, aligning with STEP-1 and STEP-5 outcomes (−14.9% and −15.2%, respectively) [29,30]. Previous studies comparing between the 2 drugs have shown mixed results, with some real-world studies reporting lower weight change outcomes [31,32]. This evaluation highlights the added value of combining medication with behavioral support by providing similar weight outcomes from completers to the mean weight loss observed in trials such as STEP-3 and SURMOUNT-3 [22,33-36].

#### Behavioral Outcomes

Over the 12-month program, notable behavior changes were observed. While fruit and vegetable intake remained stable, daily cooking declined in both cohorts—significantly so in the semaglutide group (*P*=.03). These findings could support those of other studies showing reduced energy intake on GLP-1RA therapies [37,38]. Conversely, more participants began cooking a few times a week, possibly due to program guidance, with a substantial rise in the tirzepatide group.

Physical activity also improved, particularly among initially inactive participants. The proportion of participants reporting no weekly exercise fell from 14.8% (31/209) to 6.7% (14/209; *P*=.04) in the tirzepatide cohort and from 22.3% (29/130) to 5.4% (7/130; *P*<.001) in the semaglutide cohort. While GLP-1RA–induced weight loss can include muscle loss, increased activity suggests potential benefits for body composition and mobility [39].

#### Safety and Tolerability

The side effect profiles for both semaglutide and tirzepatide were consistent with clinical trial data, with constipation, nausea, and fatigue being most common [10,29,40]. Over time, side effects decreased significantly, with more participants reporting no side effects by month 12—increasing from 41.6% (87/209) to 60.3% (126/209; *P*<.001) in the tirzepatide cohort and from 53.8% (70/130) to 67.7% (88/130) in the semaglutide cohort. Common side effects such as constipation and nausea decreased significantly in both groups (*P*<.001). These results indicate that tolerability increases and that the common side effects are often resolved with time [30,41,42].

Notably, reports of hair loss increased with time—9.6% (20/209) of the tirzepatide cohort (*P*<.001) and 5.4% (7/130) of the semaglutide cohort. This has been previously observed, although not to this degree, with most studies observing approximately a 2% increase in reporting with time [43-45].

#### Clinical and Policy Implications

Following the NICE Technology Appraisal TA1026 and NHS England’s phased rollout, ICBs are now required to prescribe tirzepatide in both primary and secondary care, whereas semaglutide remains limited to secondary care. The guidance mandates at least 9 months of wraparound care, including nutrition and behavior support, creating demand for scalable delivery models [14,16,46]. A remote service could provide cost savings of 60% to 70% in secondary care and 10% to 60% in primary care, mainly due to workforce efficiencies—although these figures are based on hypothetical service costs and modeled estimates [13,25,47].

Major implementation challenges remain, including long wait times and paused services due to capacity limits [46-48]. Integrating remote care within existing systems is essential to prevent duplication of costs. A supported *hub and spoke* model—a single point of access (hub) that is able to refer patients to the proper service (spokes; eg, tier 2-4 weight management services)—could enhance access and outcomes [48]. With remote care’s scalability, this approach could quickly reach those most in need.

### Limitations and Benefits

This 12-month study provides large-scale longitudinal evidence from 339 participants, offering substantial real-world data on remote GLP-1RA delivery effectiveness. It captures comprehensive outcomes across multiple domains, including effectiveness, safety, behavior changes, and economic impact, providing a holistic view of treatment success. Comparative insights between semaglutide and tirzepatide users reveal important differences in effectiveness and tolerability between these medications. Detailed documentation of side effects, behavioral modifications, and weight changes offers clinically relevant information for practitioners and patients considering this treatment approach.

This service evaluation exhibits self-selection bias that may limit the generalizability of the findings. Only participants who completed the full 12-month commercial intervention—recording weights and completing surveys at months 1, 3, 6, and 12—were included in the analysis. This focus on completers may overestimate treatment effectiveness as participants experiencing poor outcomes would be more likely to discontinue the service. Similarly, recruitment may limit results as the self-paying cohort likely represents a more motivated and affluent population than typical NHS patients, potentially overestimating treatment success rates. We also did not recruit for a control group, which makes addressing these selection biases, accounting for further confounding variables, and establishing true effect size difficult. The absence of clinical markers such as metabolic and cardiovascular data significantly restricts the ability to draw broader health conclusions beyond weight loss outcomes.

### Future Research Directions

Building on this study’s limitations, future research should include randomized controlled trials comparing remote and in-person delivery models, as well as long-term prospective studies to reduce bias and assess weight regain after treatment ends. Further investigation into program intensity, behavioral support, service integration, and postmedication care is also warranted.

With tirzepatide now being prescribed through NHS primary care, real-world cost-effectiveness studies are needed. These should evaluate the financial implications of widespread implementation, including primary care capacity, monitoring requirements, and long-term health outcomes, to inform sustainable commissioning decisions and optimal care pathways.

Given evidence of significant weight regain after pharmacological treatment, studying the role of behavior change in mitigating this is crucial. Coupling such research with robust financial and health economic evaluations would help determine the long-term value of these interventions and inform NHS policy and commissioning decisions.

### Conclusions

This real-world evaluation demonstrates that remotely delivered, GLP-1RA–supported weight management programs can achieve significant weight loss above and beyond corresponding medication-only clinical trial outcomes while potentially reducing health care costs by 60% to 70%. These findings have profound implications for health care delivery transformation, suggesting that digital health interventions can not only match but also surpass traditional care models in effectiveness while addressing the scalability challenges facing obesity treatment. The significant changes in weight, alongside positive behavior changes (eg, increases in cooking and exercise frequency) and changes in side effect occurrence (reduction overall over 12 months), indicate that integrated approaches combining pharmacological and behavioral support may be essential for optimizing long-term treatment success. This evidence supports a paradigm shift toward digitally enabled, behavior-focused care that could address the obesity epidemic at scale while reducing strain on traditional health care resources.

## Appendix

Multimedia Appendix 1 Health Research Authority decision tool.

## Abbreviations

GLP-1RA: glucagonlike peptide-1 receptor agonist
ICB: integrated care board
NHS: National Health Service
NICE: National Institute for Health and Care Excellence
STEP: Semaglutide Treatment Effect in People With Obesity
SWMS: specialist weight management service

## Data Availability

The datasets generated or analyzed during this study are not publicly available as participants did not consent to having their data shared but may be available from the corresponding author on reasonable request.
